# Case report: Acute kidney injury as the initial manifestation of chronic lymphocytic leukemia/small lymphocytic lymphoma

**DOI:** 10.3389/fmed.2023.1279005

**Published:** 2023-10-20

**Authors:** Sascha T. Bender, Vanja Zeremski, Thorsten Wiech, Peter R. Mertens, Christian Gross

**Affiliations:** ^1^University Clinic for Nephrology and Hypertension, Diabetes and Endocrinology, Otto-von-Guericke University, Magdeburg, Germany; ^2^University Hospital for Hematology and Oncology, Otto-von-Guericke University, Magdeburg, Germany; ^3^Institute for Pathology, University Hospital Hamburg-Eppendorf, Hamburg, Germany

**Keywords:** chronic lymphocytic leukemia/small lymphocytic lymphoma, kidney involvement, infiltration, extranodal, zanubrutinib acute kidney injury as initial manifestation of chronic lymphocytic leukemia/small lymphocytic lymphoma, paraprotein

## Abstract

Chronic lymphocytic leukemia (CLL) is a lymphoproliferative disorder often diagnosed after incidental finding of leukocytosis. Renal involvement is usually clinically silent. Symptomatic renal impairment due to CLL/small lymphocytic lymphoma (SLL) cell infiltration in the kidney tissue is uncommon, and acute kidney injury (AKI) as a presenting feature is rare. In this case report, we describe the case of a patient with AKI caused by CLL/SLL infiltration as a presenting feature. Our report highlights the possibility of kidney injury as the first evident symptom of CLL/SLL. Kidney biopsy is the mainstay in these cases in order to establish a diagnosis. Treatment with zanubrutinib resulted in improved kidney function.

## Introduction

Chronic lymphocytic leukemia (CLL) is a clonal lymphoproliferative disorder defined by the progressive accumulation of monoclonal immunocompetent B lymphocytes, primarily involving hematopoietic organs (1, 2). Although CLL and small lymphocytic lymphoma (SLL) are essentially the same diseases, the term SLL is used for cases with fewer than 5 × 10^9^/L circulating monoclonal B cells and nodal/extranodal manifestations (3).

CLL/SLL is more common in men than in women and accounts for ~30% of all types of leukemias in Western countries (4). This disease primarily affects elderly adults with a median age at diagnosis of 71 years (5).

The diagnosis of CLL is often carried out when leukocytosis is incidentally detected during routine analyses of peripheral blood cellular composition. Symptomatic presentations include complaints related to lymphadenopathy/organomegaly, recurrent infections, or classic B symptoms such as fever, weight loss, and night sweats.

B-cell CLL infiltration can affect any organ but predominantly involves lymphoid tissues. Secondary leukemic cell infiltration of the renal parenchyma, often without clinical sequela, commonly occurs in the course of CLL/SLL, as demonstrated by postmortem autopsy studies, where renal infiltration has been proven in 63–90% of all CLL/SLL cases (6–9). Acute kidney injury (AKI) incited by direct cell infiltration is uncommon (10, 11). Nie et al. and Nuguri et al. each reported only one patient with CLL/SLL and kidney injury due to associated infiltrates in recently published retrospective cohort studies of patients with lymphoproliferative disorders (12, 13). AKI as the first indication that prompts the diagnosis of CLL/SLL is extremely rare, with only seven reported cases in the literature (14–20).

Herein, we report an index patient diagnosed with AKI secondary to renal infiltration as the initial manifestation of CLL/SLL that is associated with the histopathological pattern of tubulointerstitial injury and minimal changes glomerulonephritis.

## Case presentation

A 77-year-old male Caucasian was admitted to our clinic with deterioration in kidney function alongside a weeklong history of progressive fatigue accompanied by a prior 6-month history of unintended weight loss summing up to ~8% of the total body weight. He did not report having had fever or night sweats. There were no clinical signs for infection by bacteria or viruses. His past medical history included essential arterial hypertension, chronic obstructive lung disease, classified as Global Initiative for Chronic Obstructive Lung Disease (GOLD) 1, due to former nicotine abuse without recent exacerbation, and a history of recurring pulmonary embolism. There was no past medical history of kidney disease. His regular medication, which had not changed recently before this presentation, comprised torasemide, candesartan, and amlodipine to control blood pressure, apixaban for secondary prevention of pulmonary embolism, and pantoprazole to reduce heartburn.

The patient's body temperature and blood pressure were measured at 36.7°C and 135/70 mmHg, respectively. His pulse rate and respiration were within normal limits. Fluid homeostasis was maintained. Physical examination revealed only a slightly pale complexion. Cardiorespiratory and abdominal examinations showed no abnormalities. In particular, no signs of lymphadenopathy or hepatosplenomegaly were found.

Full blood cell counts revealed normocytic normochromic anemia with hemoglobin values at 104.7 g/l [reference range 130–180 g/l] without leukocytosis or lymphocytosis. The serum biochemical analysis indicated elevated levels of creatinine of 443 μmol/l, which increased from the baseline value of 92 μmol/l over a period of ~10 weeks. Renal impairment presented with metabolic acidosis (pH 7.255) and hyperkalemia (potassium 6.1 mmol/l [ref.range 3.4–4.9 mmol/l]). The serum calcium level was measured at 2.31 mmol/l [ref.range 2.2–2.55 mmol/l]. The relevant laboratory data are presented in Table 1.

**Table 1:** Laboratory data.

**Variable**	**Reference range, adults**	**On admission**
White cell count (per μl)	3,700 9,800	9,280
Erythrocyte count (per μl)	4,600,000–6,000,000	3,380,000
Hemoglobin (g/l)	130–180	104.7
Hematocrit (%)	36.0–46.0	32%
Mean corpuscular volume (fl)	80–94	91.4
Mean corpuscular hemoglobin (fmol)	1.6–2.1	1.9
Platelet count (per μl)	150,000–375,000	232,000
Reticulocytes (%)	0.5–2.5	1.8
Differential count (%)		
Neutrophils	50–65	56
Immature granulocytes	0–5	1
Lymphocytes	20–40	29
Monocytes	2–8	9
Eosinophils	0–5	4
Basophils	0–1	1
Sodium (mmol/l)	136–145	149
Potassium (mmol/l)	3.4–4.9	6.1
Calcium (mmol/l)	2.2–2.55	2.31
C-reactive protein (mg/l)	< 5	62
Procalcitonin (ng/ml)	< 0.5	0.12
Erythrocyte sedimentation rate (mm/h)	< 20	27
Creatinine (μmol/l)	59–104	443
eGFR CKD-EPI (ml/min/1.73m^2^)	>90	10.3
Urea nitrogen (mmol/l)	3.0–9.2	7.9
Albumin (g/l)	35 52	46
Total protein (g/l)	66–87	70.5
Lactate dehydrogenase (μmol/l)	2.25–3.75	3.56
Alanine aminotransferase (μmol/l)	0.17–0.83	0.61
Aspartate aminotransferase (μmol/l)	0.17–0.85	0.33
International normalized ratio	< 1.15	1.05
Activated partial thromboplastin time (sec)	< 34.4	25.5
pH	7.37–7.45	7.255

eGFR, estimated glomerular filtration rate; CKD-EPI, Chronic Kidney Disease Epidemiology Collaboration.

Further investigations showed a marginally elevated ANA titer of 1:160, with negative results in the rest of autoimmune serology (ANCA, anti-GBM, anti-dsDNA, and ENA). Serum complement levels (C3 and C4) were quantified within the normal range. Immunoglobulin levels were within the normal range for IgA, IgG, and IgE but decreased for IgM (0.18 g/l [0.4–2.3]). Serum protein electrophoresis yielded normal results. Free light chain detection using serum specimens revealed elevated IgL-kappa level of 42 mg/l [3.3–19.4] and normal IgL-lambda with a kappa-lambda ratio of 1.62. Serum immunofixation depicted the presence of monoclonal IgG kappa. A second serum immunofixation 2 months later showed biclonal IgG kappa and lambda. Immunoglobulin levels were measured with a total IgA of 1.05 g/l [0.7–4], IgM of 0.18 g/l [0.4 2.3], and IgG of 8.83 g/l (7–16). Quantification of the clonal component could not be obtained. Infectious serology was negative for HBsAg, anti-HCV, and HIV-Ag/Ak.

The urinalysis revealed microalbuminuria with an albumine-creatinine ratio of 8.76 mg/mmol [ref. range < 2.5 mg/mmol]) and sterile leukocyturia, excluding erythrocyturia. Additionally, the urine volume indicated marked polyuria with urine output of up to 4.5 l per day.

**Figure 1:**
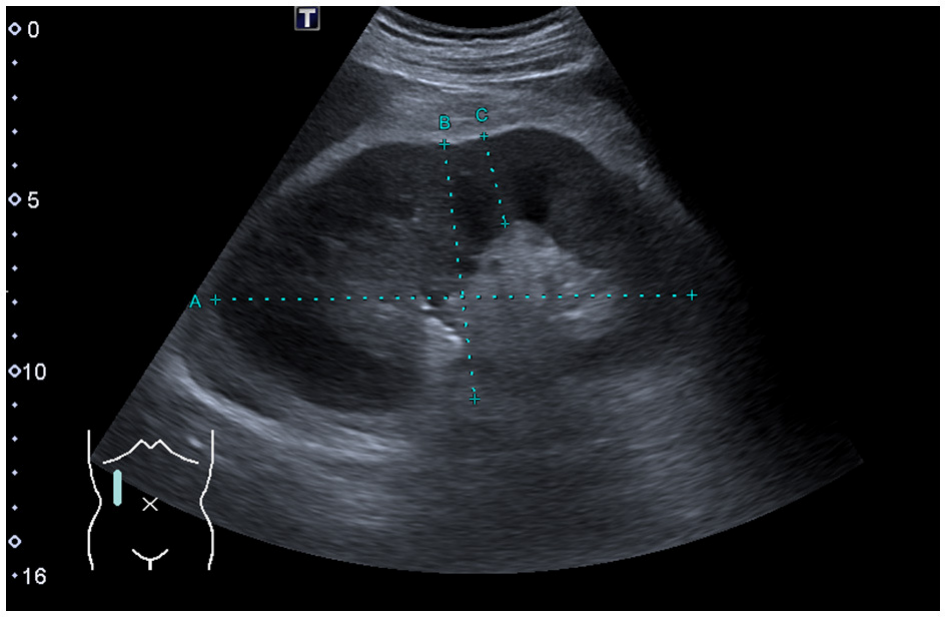
Renal ultrasound findings. Sagittal view of the right kidney showing a normal-sized kidney with swollen parenchyma measuring 26 mm (ref. range: 13–18 mm).

**Figure 2:**
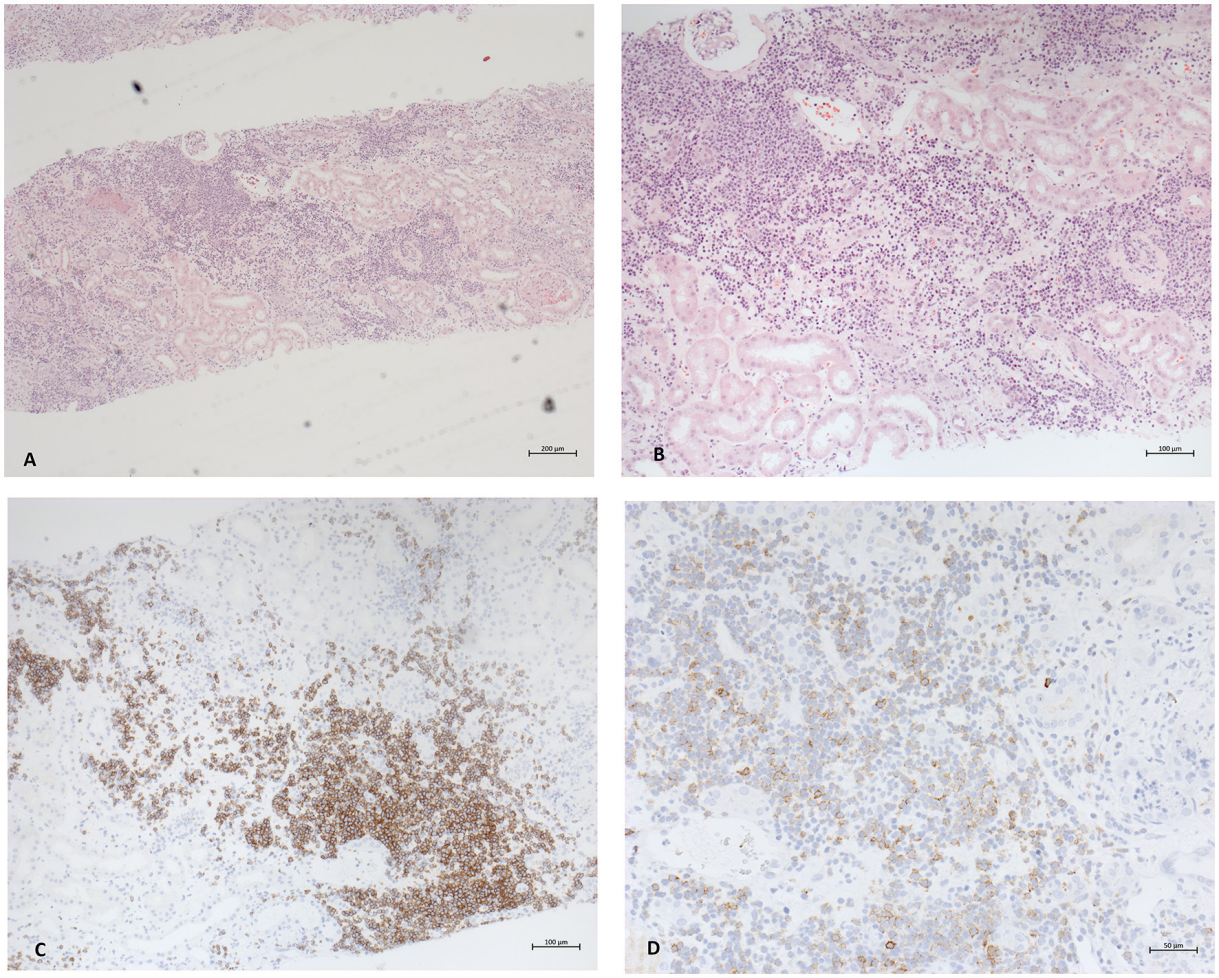
Photomicrographs of the pathological findings in renal biopsy. **(A)** Light microscopy with hematoxylin and eosin staining 5x. Diffuse dense interstitial infiltration with small lymphocyte-like cells in diffuse pattern. **(B)** Magnification depicting normal glomeruli and tubular atrophy and fibrosis alongside infiltrates (HE staining 10x). **(C, D)** Immunocytochemistry. CD20 and CD23 staining proving the presence of lymphoid B cells.

A computed tomography of the thorax demonstrated no significant lymphadenopathy. An ultrasound of the kidneys illustrated normal-sized kidneys with a swollen parenchyma and no evidence of obstructive nephropathy (Figure 1). Subsequently, a kidney biopsy was performed. Light microscopy showed dense infiltration of the cortical interstitium and capsule by mature monomorphic small lymphocytes (Figures 2A, B). Advanced tubular atrophy reached 50% of the cortical tissue surrounded by interstitial fibrosis. Immunostaining was negative for immunoglobulins and 1+ positive for complement (C3 and C1q). Immunocytochemical staining revealed that the infiltrate consisted mainly of monoclonal B lymphoid cells, which tested positive for CD20 and CD23 (Figures 2C, D). Additionally, aberrant expression of CD5 was observed. The glomeruli showed only minimal changes, as can be observed through electron microscopy, characterized by a global loss of podocyte foot processes.

Given the biopsy findings, the patient was referred to the clinic for hematology for further evaluation. There was no evidence of hemolysis. Flow cytometry of peripheral blood confirmed the presence of a CD5 positive monotypic B-cell population expressing CD19, CD20, CD23, and IgM and exhibiting kappa light chain restriction. However, criteria of CLL were not met (monoclonal B cell count < 5 × 10^9^/L). Further analysis showed an immunoglobulin heavy chain (IgHV) mutated status and absence of TP53 mutation/deletion. Bone marrow examination displayed typical monomorphic infiltration in up to 40% of the medullary cavity, with an immunophenotype consistent with CLL/SLL, but without infiltration of plasma cells, ruling out the differential diagnosis of multiple myeloma.

In summary, all the findings clearly present a diagnosis of CLL/SLL. The overall condition of the patient was satisfactory, with an Karnofsky performance score of 80. Treatment with the selective Bruton tyrosine kinase inhibitor, zanubrutinib, at a dosage of 160 mg twice daily together with prednisolone 50 mg, due to the described distinct minimal change glomerulonephropathy pattern, was initiated. Potential nephrotoxic medications including candesartan were temporarily discontinued. Kidney function recovered, electrolytes and metabolism normalized, and polyuria and proteinuria resolved. Moreover, renal replacement therapy was not necessary. At the last follow-up 6 month later, kidney function remained stable with a creatinine level of 170 μmol/l. No clinically visible side effects under zanubrutinib have been observed to date. The overall clinical condition continued to be moderate to good.

## Discussion

In previous studies, CLL/SLL-associated kidney disease remains poorly described, thus limiting our understanding. This is primarily due to the infrequent occurrence of kidney biopsy in patients with CLL/SLL (21).

Previous reports depicted a wide spectrum of clinical presentation and histopathological patterns. Patient characteristics and the clinical course of reported cases with AKI resulting from B-cell infiltration as a presenting feature in undiagnosed CLL/SLL are compiled in Table 2.

**Table 2:** Patient characteristics and clinical parameters of reported CLL/SLL cases initially presenting with kidney failure.

**Reference**	**Sex**	**Age (years)**	**Lymphadenopathy**	**White cell count (per μl)**	**Lymphocyte count (per μl)**	**Hemoglobin (mmol/l)**	**Platelet count (per μl)**	**Creatinine (μmol/l)**	**Proteinuria (mg/day)**	**Requiring dialysis**
Kayer et al. (14)	M	64	–	18,600	12,090	4.84	265,000	194.48	100	–
Uprety et al. (15)	M	70	+	16,200	9.136	8.32	198,000	1,343.68	NA	+
Dou et al. (16)	M	54	+	16,800	10,382	Normal	NA	290.04	5,000	–
Hewamana et al. (17)	M	55	+	19,000	10,000	7.07	19,000	969	Mild	–
Erten et al. (18)	F	73	+	13,000	5,600	7.14	139,000	362	Mild	–
Tucker et al. (19)	M	70	+	124,000	114,080	6.21	159,000	1,200	270	+
Saggi et al. (20)	M	65	+	186,000	167,400	NA	180,000	1,478.4	NA	+

AKI, acute kidney injury; M, male; F, female; NA, not available.

Eight cases, including the presented one, were diagnosed with CLL/SLL for the first time through kidney biopsy. Among these cases, seven patients were men (87.5 %), and six (75%) out of eight patients were younger than the median age at a diagnosis of 71 years. To date, all reported cases have indicated peripheral leukocytosis at the time of diagnosis. Mild proteinuria is observed in six cases. Lymphocyte counts and renal impairment, characterized by an increase in serum creatinine levels and proteinuria, did not exhibit correlation. Additionally, the degree of AKI had no correlation with the stage of CLL/SLL. Nevertheless, Wang et al. demonstrated that severe renal insufficiency determined as serum creatinine levels equaling 200 μmol/l or more is associated with ≥ 50% interstitial infiltration, which coincides with our data (22). Kidney enlargement may be present, but this finding is inconsistent and is of low sensitivity and specificity (23–25). Pathomechanistically, it remains unclear how exactly CLL/SLL infiltration causes kidney injury. It has been postulated that infiltration may cause compression of both the tubular system and microvasculature inducing intrarenal obstruction and ischemia (26, 27). However, in most case reports, no such findings could be described. In the biopsy specimen of our index patient, no evidence supporting this theory could be observed (Figure 2). Schwartz et al. reported the accompanying fibrosis, particularly in areas of leukemic infiltration, and postulated an infiltration-associated inflammatory mechanism (8). In accordance with this theory of direct harm are the findings of Wang et al. verifying that CLL/SLL cells can secrete monoclonal immunoglobulins *in situ* (22).

Usually, infiltration of the renal interstitium is diffuse, occasionally nodular, and develops in the later stage of the illness (28, 29). Typically, it is associated with both glomerular lesions, with membranoproliferative- and minimal change-like patterns appearing to be the most frequent, alongside secondary tubular atrophy (8, 10, 21). It may even directly affect the tubules in selected cases (19, 30, 31). Nevertheless, Corlu et al. and Strati et al. found, independently, an almost even distribution between diffuse and focal infiltration patterns with no difference in renal function among these groups (21, 25). Our patient exhibited diffuse infiltration of the atrophic tubulointerstitium and displayed glomerular minimal changes. However, the latter finding despite the pronounced advanced loss of foot processes in our specimen did not correspond with the clinical presentation, since there was only mild proteinuria detected by urinalysis without clinical signs of nephrotic syndrome, ruling out minimal change disease in our patient.

Administered treatment with zanubrutinib in this case followed the SEQUOIA-trial, recently establishing zanubrutinib as a novel treatment option for untreated CLL/SLL, showing significantly improved progression-free survival rates (32). To our knowledge, we herein describe the first case showing that zanubrutinib could contribute to stabilization of kidney function in a CLL/SLL patient presenting initially with B-cell infiltration-associated acute kidney injury.

Detection of clonal paraprotein, similar to our case, can be found frequently in patients with CLL/SLL and may even precede the diagnosis (33–35). Mozas et al. reported a biclonal serum immunofixation to be detectable in 11% of the cases in a single center study (36). As the origin of this phenomenon is not clear, there are different interpretations of this observation, including clonal evolution and the occurrence of isotype switching (36). Nevertheless, the presence of this feature is associated with poorer overall survival, with biclonal cases appearing to have a worse prognosis (33, 36).

AKI is known to be associated with a less lasting hematological response (37). Kidney disease is a well-established negative prognostic factor for overall survival in CLL/SLL patients (38). Being a major cause of morbidity and mortality, it is important to address renal function to improve long-term outcomes, especially considering that adequate treatment of the hematologic primary disease can improve the kidney function (24, 28, 39–41). For this reason, diagnostic security is to be achieved quickly through kidney biopsy, which is often unavoidable, similar to the index case at hand, for early treatment initiation.

## Conclusion

B-cell infiltration should be considered despite its rare clinical manifestation as differential diagnosis of AKI in patients with CLL/SLL. AKI may present as the first clinical feature of CLL/SLL as highlighted in our index case, detectable only after kidney biopsy, which is mandatory in these cases to establish diagnosis and early treatment. Treatment with zanubrutinib can help to improve the kidney function. Close interdisciplinary cooperation between nephrologists and hematooncologists is crucial in managing these patients.

## Data availability statement

The original contributions presented in the study are included in the article/supplementary material, further inquiries can be directed to the corresponding author.

## Ethics statement

Written informed consent was obtained from the individual(s) for the publication of any potentially identifiable images or data included in this article.

## Author contributions

SB: Conceptualization, Data curation, Writing—original draft, Writing—review and editing. VZ: Writing—review and editing. TW: Visualization, Writing—review and editing. PM: Conceptualization, Supervision, Writing—review and editing. CG: Supervision, Writing—review and editing.

## References

[1] Klein U Dalla-Favera R. New insights into the pathogenesis of chronic lymphocytic leukemia. Sem Cancer Biol. (2010) 20:377–83. 10.1016/j.semcancer.2010.10.012 21029776

[2] Hallek M Cheson BD Catovsky D Caligaris-Cappio F Dighiero G Döhner H. iwCLL guidelines for diagnosis, indications for treatment, response assessment, supportive management of CLL. Blood. (2018) 131:2745–60. 10.1182/blood-2017-09-806398 29540348

[3] Weltgesundheitsorganisation. WHO Classification of Tumours of Haematopoietic and Lymphoid Tissues. Revised 4th edition. In:Swerdlow SH Campo E Harris NL, editors. International Agency for Research on Cancer (2017).

[4] Siegel RL Miller KD Wagle NS Jemal A. Cancer statistics, 2023. CA A Cancer J Clinic. (2023) 73:17–48. 10.3322/caac.21763 36633525

[5] Smith A Howell D Patmore R Jack A Roman E. Incidence of haematological malignancy by sub-type: a report from the Haematological malignancy research network. Br J Cancer. (2011) 105:1684–92. 10.1038/bjc.2011.450 22045184 PMC3242607

[6] Barcos M Lane W Gomez GA Han T Freeman A Preisler H. An autopsy study of 1206 acute and chronic leukemias (1958 to 1982). Cancer. (1987) 60:827–37. 3474054 10.1002/1097-0142(19870815)60:4<827::aid-cncr2820600419>3.0.co;2-a

[7] Xiao JC Walz-Mattmüller R Ruck P Horny HP Kaiserling E. Renal involvement in myeloproliferative and lymphoproliferative disorders: a study of autopsy cases. Gen Diagn Pathol. (1997) 142:147–53. 9065578

[8] Schwartz JB Shamsuddin AM. The effects of leukemic infiltrates in various organs in chronic lymphocytic leukemia. Human Pathol. (1981) 12:432–40. 10.1016/S0046-8177(81)80023-8 7250955

[9] Kirshbaum JD. Leukemia: a clinical and pathologic study of one hundred and twenty-three fatal cases in a series of 14,400 necropsies. Arch Intern Med (Chic). (1943) 71:777. 10.1001/archinte.1943.00210060038003

[10] Kowalewska J Nicosia RF Smith KD Kats A Alpers CE. Patterns of glomerular injury in kidneys infiltrated by lymphoplasmacytic neoplasms. Human Pathol. (2011) 42:896–903. 10.1016/j.humpath.2010.09.009 21288559

[11] Da'as N Polliack A Cohen Y Amir G Darmon D Kleinman Y. Kidney involvement renal manifestations in non-Hodgkin's lymphoma lymphocytic leukemia: a retrospective study in 700 patients: Renal involvement in CLL NHL. Euro J Haematol. (2001). 67(3):158-164. 10.1034/j.1600-0609.2001.5790493.x 11737248

[12] Nie G Sun L Zhang C. Clinicopathological features and individualized treatment of kidney involvement in B-cell lymphoproliferative disorder. Front Immunol. (2022) 13:903315. 10.3389/fimmu.2022.903315 36172352 PMC9510618

[13] Nuguri S Swain M De Padua M Gowrishankar S. Renal lymphoma diagnosed on kidney biopsy presenting as acute kidney injury. Indian J Nephrol. (2022) 32:342. 10.4103/ijn.ijn_345_21 35967526 PMC9364996

[14] Kayar Y Ekinci I Bay I Bayram Kayar N Hamdard J Kazancioglu R. Acute renal failure due to Leukaemic infiltration in chronic lymphocytic leukaemia. Case Rep Med. (2015) 2015:1–3. 10.1155/2015/469136 PMC4471381 26146503

[15] Uprety D Peterson A Shah BK. Renal failure secondary to leukemic infiltration of kidneys in CLL—a case report and review of literature. Ann Hematol. (2013) 92:271–3. 10.1007/s00277-012-1547-6 22875063

[16] Dou X Hu H Ju Y Liu Y Kang K Zhou S. Concurrent nephrotic syndrome and acute renal failure caused by chronic lymphocytic leukemia (CLL): a case report and literature review. Diagn Pathol. (2011) 6:99. 10.1186/1746-1596-6-99 21995711 PMC3206418

[17] Hewamana S Pepper C Jenkins C Rowntree C. Acute renal failure as the presenting feature of leukaemic infiltration in chronic lymphocytic leukaemia. Clin Exp Nephrol. (2009) 13:179–81. 10.1007/s10157-009-0129-y 19255826

[18] Erten N Saka B Caliskan YK Besisik S Karan MA Tascioglu C. Acute renal failure due to leukaemic infiltration in chronic lymphocytic leukaemia: case report: acute renal failure due to leukaemic infiltration. Int J Clinic Pract. (2005) 59:53–5. 10.1111/j.1368-504X.2005.00049.x 15875623

[19] Tucker B Brown AL d'Ardenne AJ Cattell WR. Reversible renal failure due to renal infiltration and associated tubulointerstitial disease in chronic lymphocytic leukaemia. Nephrology Dialysis Transplantation. (1990) 5:616–8. 10.1093/ndt/5.8.616 23275997

[20] Saggi S Calandri C Muhlfelder T Choi H Kahn T Kaji D. Renal failure due to leukaemic infiltration in chronic lymphocytic leukaemia. Nephrol Dial Transplant. (1990) 5:1051–2. 10.1093/ndt/5.12.1051 2128953

[21] Strati P Nasr SH Leung N Hanson CA Chaffee KG Schwager SM. Renal complications in chronic lymphocytic leukemia and monoclonal B-cell lymphocytosis: the Mayo Clinic experience. Haematologica. (2015) 100:1180–8. 10.3324/haematol.2015.128793 26088927 PMC4800708

[22] Wang H Yu X Zhang X Wang S Zhao M. The pathological features of leukemic cells infiltrating the renal interstitium in chronic lymphocytic leukemia/small lymphocytic lymphoma from a large single Chinese center. Diagn Pathol. (2021) 16:59. 10.1186/s13000-021-01120-4 34218814 PMC8254985

[23] Bach AG Behrmann C Holzhausen HJ. Prevalence and patterns of renal involvement in imaging of malignant lymphoproliferative diseases. Acta Radiol. (2012) 53:343–8. 10.1258/ar.2011.110523 22287149

[24] Poitou-Verkinder A-L Francois A Drieux F Lepretre S Legallicier B Moulin B. The Spectrum of Kidney Pathology in B-Cell Chronic Lymphocytic Leukemia/Small Lymphocytic Lymphoma: A 25-Year Multicenter Experience. Rastaldi MP, ed. PLoS ONE. (2015). 10:e0119156. 10.1371/journal.pone.0119156 25811382 PMC4374947

[25] Corlu L Rioux-Leclercq N Ganard M Decaux O Houot R Vigneau C. Renal dysfunction in patients with direct infiltration by B-Cell Lymphoma. Kidney Int Rep. (2019) 4:688–697. 10.1016/j.ekir.2019.02.008 31080924 PMC6506703

[26] Obrador GT Price B O'Meara Y Salant DJ. Acute renal failure due to lymphomatous infiltration of the kidneys. J Am Soc Nephrol. (1997) 8:1348–54. 10.1681/ASN.V881348 9259365

[27] Junglee N Shrikanth S Seale J. Rapidly progressive renal failure due to chronic lymphocytic leukemia—response to chlorambucil. Indian J Nephrol. (2012) 22:217. 10.4103/0971-4065.98766 23087560 PMC3459529

[28] Ferreira AC Brum S Carvalho D Pataca I Carvalho F Santos MC. Renal dysfunction due to leukemic infiltration of kidneys in a case of chronic lymphocytic leukemia. Hemodialysis Int. (2010) 14:87–90. 10.1111/j.1542-4758.2009.00395.x 19758305

[29] Norris HJ Wiener J. The renal lesions in leukemia. Am J Med Sci. (1961) 241:512–8. 10.1097/00000441-196104000-00016 13729602

[30] Sangala N Dewdney A Marley N Cranfield T Venkat-Raman G. Progressive renal failure due to renal infiltration by BK polyomavirus and leukaemic cells: which is the culprit? Clinical Kidney J. (2011) 4:46–8. 10.1093/ndtplus/sfq193 25984102 PMC4421626

[31] Rockx MA Rizkalla K Clark WF. Acute renal failure and chronic lymphocytic leukaemia. Nephrol Dial Transplant. (2007) 23:770–1. 10.1093/ndt/gfm617 17998226

[32] Tam CS Brown JR Kahl BS Ghia P Giannopoulos K Jurczak W. Zanubrutinib versus bendamustine and rituximab in untreated chronic lymphocytic leukaemia and small lymphocytic lymphoma (SEQUOIA): a randomised, controlled, phase 3 trial. Lancet Oncol. (2022) 23:1031–43. 10.1016/S1470-2045(22)00293-5 35810754

[33] Xu W Wang YH Fan L. Prognostic significance of serum immunoglobulin paraprotein in patients with chronic lymphocytic leukemia. Leukemia Res. (2011) 35:1060–5. 10.1016/j.leukres.2010.12.005 21208658

[34] Tsai HT Caporaso NE Kyle RA. Evidence of serum immunoglobulin abnormalities up to 9.8 years before diagnosis of chronic lymphocytic leukemia: a prospective study. Blood. (2009) 114:4928–32. 10.1182/blood-2009-08-237651 19828698 PMC2788972

[35] Beaume A Brizard A Dreyfus B Preud'homme JL. High incidence of serum monoclonal Igs detected by a sensitive immunoblotting technique in B-cell chronic lymphocytic leukemia. Blood. (1994) 84:1216–9. 8049436

[36] Mozas P Pineyroa JA Nadeu F Magnano L Rivero A Rivas-Delgado A. Serum monoclonal component in chronic lymphocytic leukemia: baseline correlations and prognostic impact. Haematol. (2020) 106:1754–7. 10.3324/haematol.2020.263228 33147939 PMC8168485

[37] Canet E Zafrani L Lambert J Thieblemont C Galicier L Schnell D. Acute Kidney Injury in Patients with Newly Diagnosed High-Grade Hematological Malignancies: Impact on Remission and Survival. PLoS ONE. (2013) 8:e55870. 10.1371/journal.pone.0055870 23457485 PMC3573047

[38] Strati P Chaffee KG Achenbach SJ Slager SL Leung N Call TG. Renal insufficiency is an independent prognostic factor in patients with chronic lymphocytic leukemia. Haematologica. (2017) 102:e22–5. 10.3324/haematol.2016.150706 27634202 PMC5210254

[39] Comerma-Coma M. Reversible renal failure due to specific infiltration of the kidney in chronic lymphocytic leukaemia. Nephrol Dialysis Transplant. (1998) 13:1550–2. 10.1093/ndt/13.6.1550 9641192

[40] Pagniez DC Fenauxaaa P Delvallez L Dequiedt P Gosselin B Tacquet A. Reversible renal failure due to specific infiltration in chronic lymphocytic leukemia. Am J Med. (1988) 85:579–80. 10.1016/S0002-9343(88)80105-0 3177414

[41] Phillips JK Bass PS Majumdar G Davies DR Jones NF Pearson TC. Renal failure caused by leukaemic infiltration in chronic lymphocytic leukaemia. J Clinic Pathol. (1993) 46:1131–3. 10.1136/jcp.46.12.1131 8282840 PMC501728

